# Plasma potassium, diuretic use and risk of developing chronic kidney disease in a predominantly White population

**DOI:** 10.1371/journal.pone.0174686

**Published:** 2017-03-27

**Authors:** Lyanne M. Kieneker, Michele F. Eisenga, Michel M. Joosten, Rudolf A. de Boer, Ron T. Gansevoort, Jenny E. Kootstra-Ros, Gerjan Navis, Stephan J. L. Bakker

**Affiliations:** 1 Department of Internal Medicine, University of Groningen, University Medical Center Groningen, Groningen, the Netherlands; 2 Department of Cardiology, University of Groningen, University Medical Center Groningen, Groningen, the Netherlands; 3 Department of Laboratory Medicine, University of Groningen, University Medical Center Groningen, Groningen, the Netherlands; The University of Tokyo, JAPAN

## Abstract

**Objective:**

Both hypokalemia and hyperkalemia are associated with disease progression in patients with chronic kidney disease (CKD). It is unclear whether similar associations are present in the general population. Our aim was to examine the association of plasma potassium with risk of developing CKD and the role of diuretics in this association in a population-based cohort.

**Research design and methods:**

We studied 5,130 subjects free of CKD at baseline of the Prevention of Renal and Vascular End-Stage Disease (PREVEND) study, a prospective, population-based cohort of Dutch men and women aged 28–75 years. Hypokalemia was defined as plasma potassium <3.5 mmol/L, and hyperkalemia as plasma potassium ≥5.0 mmol/L. Risk of CKD was defined as *de novo* development of eGFR <60 ml/min/1.73m^2^ and/or albuminuria >30 mg/24h.

**Results:**

Mean baseline plasma potassium was 4.4±0.3 mmol/L. The prevalences of hypokalemia and hyperkalemia were 0.5% and 3.8%, respectively; 3.0% of the subjects used diuretics. During a median follow-up of 10.3 years (interquartile range: 6.3–11.4 years), 753 subjects developed CKD. The potassium-CKD association was modified by diuretic use (P_interaction_ = 0.02). Both hypokalemia without (HR, 7.74, 95% CI, 3.43–17.48) or with diuretic use (HR, 4.32, 95% CI, 1.77–10.51) were associated with an increased CKD risk as compared to plasma potassium 4.0–4.4 mmol/L without diuretic use. Plasma potassium concentrations ≥3.5 mmol/L were associated with an increased CKD risk among subjects using diuretics (P_trend_ = 0.01) but not among subjects not using diuretics (P_trend_ = 0.74).

**Conclusion:**

In this population-based cohort, hypokalemia was associated with an increased CKD risk, regardless of diuretic use. In the absence of hypokalemia, plasma potassium was not associated with an increased CKD risk, except among subjects using diuretics.

## Introduction

Potassium homeostasis is strictly regulated by the kidneys and plasma potassium is normally maintained within narrow limits (typically between 3.5 to 5.0 mmol/L) [1–3]. Such strict regulation of plasma potassium is essential for many physiologic processes, including acid-base homeostasis, systemic blood pressure control, smooth muscle tone, cardiac conduction and rhythm, and membrane potential repolarization [1].

Disturbances in plasma potassium are more common in patients with chronic kidney disease (CKD) compared to the general population. This disturbance typically presents as hypokalemia (<3.5 mmol/L) as a consequence of diuretic administration [4], but CKD patients also have a higher risk of having hyperkalemia (≥5.0 mmol/L) due to impaired kidney function and frequent use of renin angiotensin aldosterone system (RAAS) inhibitors [1]. In subjects with CKD, both hypo- and hyperkalemia are associated with higher risk of all-cause mortality, major cardiovascular events, and hospitalization [5–7]. Also in individuals without impaired kidney function, potassium disturbances are linked to higher rates of mortality [8]. Moreover, in experimental studies, it has been shown that hypokalemia can induce renal injury [9,10]. Prospective cohort studies in CKD patients showed that hypokalemia is associated with increased risk of developing of end-stage renal disease (ESRD) in most [7,11,12], but not all studies [6].

The association of hypokalemia with risk of developing *de novo* CKD is not well established. A study in Japanese men and women examined this association and found that potassium <4.0 mmol/L was associated with an increased risk of developing CKD [13]. However, this study excluded individuals using medication for hypertension including diuretics–the main risk factor for hypokalemia in the general population [4]–which substantially limits the generalization of the findings. Recently, Chen at al. observed that lower levels of potassium were associated with higher CKD risk among participants of the Atherosclerosis Risk in Communities Study not taking thiazide and loop diuretics [14].

Therefore, the aim of the present study was to prospectively examine the association of plasma potassium with risk of developing CKD in a Dutch large population-based cohort and to investigate the role of diuretic use in this association.

## Materials and methods

### Study design and population

This study was conducted within the framework of the PREVEND study, which is designed to prospectively investigate the natural course of increased levels of urinary albumin excretion (UAE) and its relation to renal and cardiovascular disease in a large cohort drawn from the general population. Details of this study have been described elsewhere [15]. In brief, from 1997 to 1998, all inhabitants of Groningen, the Netherlands aged 28 to 75 years, were sent a questionnaire and a vial to collect a first morning void urine sample. Pregnant women and subjects with type 1 diabetes mellitus were excluded. Urinary albumin concentration was assessed in 40,856 responders. Subjects with a urinary albumin concentration of ≥10 mg/L (n = 7,768) were invited to participate, of whom 6,000 were enrolled. In addition, a randomly selected group with a urinary albumin concentration of <10 mg/L (n = 3,394) was invited to participate in the cohort, of whom 2,592 were enrolled. These 8,592 individuals form the Prevention of Renal and Vascular End-stage Disease (PREVEND) cohort and completed an extensive examination in 1997 and 1998 (baseline).

For the current study, we excluded subjects with CKD at baseline (n = 1,898) or unknown CKD status (n = 1,191), with missing values of plasma potassium due to missing blood samples or insufficient volume of plasma (n = 358), or invalid concentrations of plasma potassium (n = 8), and with renal disease requiring dialysis (n = 7), leaving 5,130 participants for the analyses.

The PREVEND study has been approved by the medical ethics committee of the University Medical Center Groningen and is performed in accordance with Declaration of Helsinki guidelines. Written informed consent was obtained from all participants.

### Data collection

The procedures at each examination in the PREVEND study have been described in detail previously [16]. In brief, each examination included two visits to an outpatient clinic separated by three weeks. Prior to the first visit, all participants completed a self-administered questionnaire regarding demographics, cardiovascular and renal disease history, smoking habits, alcohol consumption and medication use. Information on medication use was combined with information on drug use from the IADB.nl database, containing pharmacy-dispensing data from community pharmacies in the Netherlands [17]. During each examination and during each visit, blood pressure was measured on the right arm, every minute for 10 and 8 minutes, respectively, by an automatic Dinamap XL Model 9300 series device (Johnson-Johnson Medical, Tampa, Florida, USA). The mean of the last two recordings from each of the two visits was used. In the last week before the second visit, subjects collected two consecutive 24-hour specimens after thorough oral and written instruction. Furthermore, fasting serum and plasma blood samples were provided and stored at -80°C.

### Potassium measurement and categories

Circulating potassium was determined in plasma with lithium heparin as anticoagulant on a Modular analyzer (Roche Diagnostics, Mannheim, Germany) with an interassay coefficient of variation of 1.4%. Plasma potassium was categorized as <3.5, 3.5–3.9, 4.0–4.4, 4.5–4.9, and ≥5.0 mmol/L. Hypokalemia was defined as a plasma potassium concentration less than 3.5 mmol/L, normokalemia was defined as a potassium concentration between 4.0 and 4.4 mmol/L, and hyperkalemia was defined as a plasma potassium concentration equal to or greater than 5.0 mmol/L.

### Ascertainment of chronic kidney disease

The primary outcome of CKD was defined as a combination of reaching an eGFR <60 ml/min per 1.73 m^2^ and/or an UAE >30 mg/24h *de novo*. We estimated GFR with the combined creatinine cystatin C-based Chronic Kidney Disease Epidemiology (CKD-EPI) Collaboration equation from 2012, taking into account age, sex, and race [18]. UAE was multiplied by urine volume to obtain a value in mg per 24h. The two 24-hour UAE values of each subject per examination were averaged. Data on both measurements were collected during follow-up every 3 to 5 years.

Measurement of serum creatinine was performed by an isotope dilution mass spectrometry (IDMS) traceable enzymatic method on a Roche Modular analyzer using reagents and calibrators from Roche (Roche Diagnostics, Mannheim, Germany), with intra- and interassay coefficients of variation of 0.9% and 2.9%, respectively. Serum cystatin C concentrations were measured by Gentian Cystatin C Immunoassay (Gentian AS, Moss, Norway) on a Modular analyzer (Roche Diagnostics). Cystatin C was calibrated directly using the standard supplied by the manufacturer (traceable to the International Federation of Clinical Chemistry Working Group for Standardization of Serum Cystatin C) [19]. The intra- and interassay coefficients of variation were <4.1% and <3.3%, respectively. Urinary albumin concentration was measured by nephelometry with a threshold of 2.3 mg/l, and intra- and interassay coefficients of variation of 2.2% and 2.6%, respectively (Dade Behring Diagnostic, Marburg, Germany).

### Assessment of covariates

BMI was calculated as weight (kg) divided by height squared (m^2^). Smoking status was defined as self-reported never smoker, former smoker, or current smoker (<6, 6–20, or >20 cigarettes/day) and alcohol intake as no/rarely, 1 to 4 drinks/month, 2 to 7 drinks/week, 1 to 3 drinks/day, and 4 or more drinks/day. Parental history of CKD was defined as having a first-degree relative who had a renal disease requiring dialysis for >6 weeks. Educational level was defined as low (primary education or intermediate vocational education), middle (higher secondary education), and high (higher vocational education and university). Use of diuretic medication was defined as use of low-ceiling diuretics including thiazides (ATC code C03A), low-ceiling diuretics excluding thiazides (ATC code C03B), high-ceiling diuretics (ATC code C03C), potassium-sparing agents (ATC code C03D), and diuretics and potassium-sparing agents in combination (ATC code C03E).

Urinary excretions of sodium, potassium, magnesium, and creatinine were determined with a MEGA clinical chemistry analyzer (Merck, Darmstadt, Germany). Sodium, potassium, and magnesium were determined by indirect potentiometry. Glucose was determined from fasting blood samples by using Kodak Ektachem dry chemistry (Eastman Kodak, Rochester, NY). High-sensitivity C-reactive protein was determined by nephelometry (BN II, Dade Behring, Marburg, Germany). Concentrations of 1,25-dihydroxyvitamin D were measured using liquid chromatography tandem mass spectrometry as previously described [20]. An automated two-site immunoassay (Roche, diagnostics, Indianapolis, IN, USA) was used to measure plasma intact parathyroid hormone (PTH). Measurement of blood urea nitrogen (BUN) was performed on a Roche Modular with ultraviolet kinetic assay, which is based on Talke and Schubert’s method and has been optimized for analyzers that permit kinetic measurements.

Type 2 diabetes was defined as a fasting plasma glucose ≥7.0 mmol/L (>126 mg/dL) or the use of glucose-lowering drugs [21]. Hypertension was defined as systolic blood pressure of ≥140 mm Hg, a diastolic blood pressure of ≥90 mm Hg, or both or the use of antihypertensive agents as previously described [22].

### Statistical analyses

Baseline characteristics are presented according to categories of plasma potassium. Continuous data are presented as mean with SD or as median with IQR in case of skewed distribution. Categorical data are presented as number with percentage.

Multinomial logistic regression was used to examine the associations of the predefined risk factors with the plasma potassium categories. Plasma potassium of 4.0–4.4 mmol/L was used as reference category. ORs are reported with 95% CIs. Univariable associations with risk of hypo- and hyperkalemia were determined for sex, age, BMI, eGFR, smoking, alcohol, education, race, diabetes, hypertension, use of ACEi, ARBs, beta blockers, thiazide diuretics, loop diuretics, potassium-sparing diuretics, and urinary excretion of potassium, magnesium and albumin. These analyses were also adjusted for eGFR for bivariable ORs (95% CIs).

To study the prospective association between plasma potassium and risk of developing CKD, Cox proportional hazards regression analyses were used to calculate HRs and 95% CIs. We first calculated HRs for the crude model. Second, we additionally adjusted for age, sex, and baseline eGFR. Third, we additionally adjusted for height, weight, urinary potassium excretion, and use of diuretics. Finally, we additionally adjusted for systolic blood pressure, plasma aldosterone, plasma albumin, plasma magnesium [23], hs-CRP, diabetes, smoking, alcohol, education, race, urinary creatinine excretion, plasma chloride, and the BUN/creatinine ratio one at the time. In secondary analyses, CKD incidence was defined by either an eGFR <60 ml/min/1.73 m^2^ or UAE >30 mg/24h alone. We evaluated potential effect modification by sex, age, hypertension, urinary potassium excretion, baseline eGFR, plasma aldosterone, and diuretic use by fitting models containing both main effects and their cross-product terms.

Several sensitivity analyses were performed to examine the robustness of the associations between plasma potassium and risk of developing CKD. First, we examined the specific role of potassium-wasting diuretics (i.e. loop diuretics and thiazide diuretics) in the association of plasma potassium and risk of developing CKD. Second, we excluded subjects at baseline with an eGFR <66 (instead of <60) ml/min per 1.73 m^2^ and/or a UAE >25 (instead of >30) mg/24h for a more pronounced decline in kidney function over time in order to reach the primary outcome of CKD. Third, we restricted the analyses of plasma potassium and risk of CKD to subjects who were not using antihypertensive drugs at baseline because of the potential mediating effects of blood pressure in the association between plasma potassium and risk of CKD. Fourth, we excluded subjects from the analyses who developed diabetes during follow-up to eliminate the development of diabetes during follow-up as possible mediator in the association of plasma potassium with risk of developing CKD. Finally, we addressed the oversampling of subjects with higher urinary albumin concentrations by using design-based Cox proportional-hazards regression models that took into account the probability of selection by statistical weighting.

All P-values are two-tailed. A P-value <0.05 was considered statistically significant. All analyses were conducted with the use of the statistical package IBM SPSS (version 22; SPSS, Chicago, IL) and Rstudio (version 0.99.491; Rstudio, Boston, MA).

## Results

### Baseline characteristics

**Fig 1:**
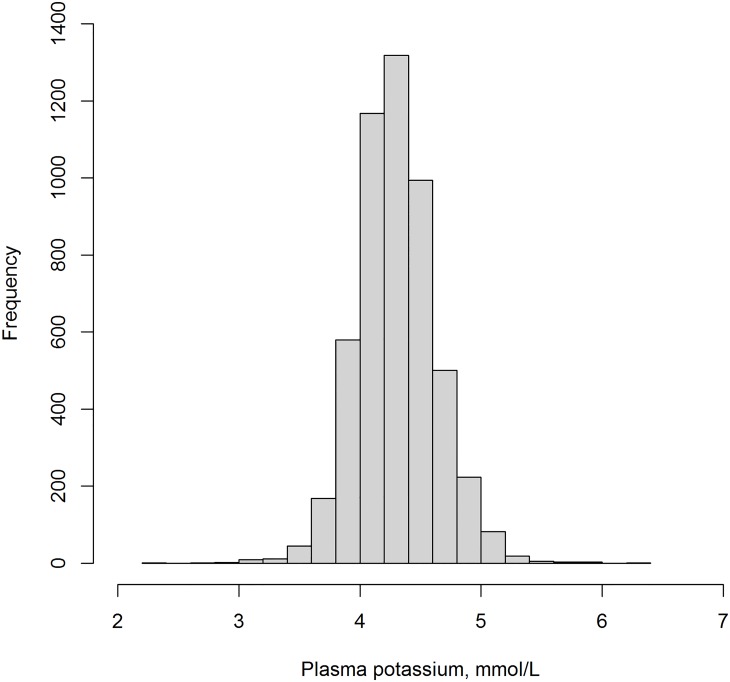
Distribution of plasma potassium in 5,130 subjects of the Prevention of Renal and Vascular End-Stage Disease (PREVEND) study.

The distribution of plasma potassium is presented in Fig 1. Baseline mean plasma potassium was 4.4±0.3 mmol/L. The prevalence of hypokalemia at baseline was 0.5%. Hyperkalemia was more common with a prevalence of 3.8%. A total of 3.0% of the subjects used diuretics, including thiazides (1.8%), loop diuretics (0.4%), and potassium-sparing diuretics (0.8%).

Baseline characteristics according to categories of plasma potassium are shown in Table 1. Subjects with hypokalemia were more likely to be older, to be lower educated, not smoking, consume no alcohol, and to have diabetes compared to subjects with normokalemia. They were also more likely to use beta blockers and diuretics, and had a higher systolic blood pressure and lower plasma chloride concentrations compared to subjects with normokalemia. Furthermore, a slightly lower eGFR and slightly higher UAE were observed in subjects with hypokalemia. Subjects with hyperkalemia were more likely to be male, to smoke, to be White, have a higher urinary potassium excretion, and to not use angiotensin-converting-enzyme inhibitors (ACEi), angiotensin receptor blockers (ARBs), and thiazide diuretics compared to subjects with normokalemia.

**Table 1:** Baseline characteristics of the 5,130 participants of the Prevention of Renal and Vascular End-Stage Disease (PREVEND) study.

	Plasma potassium, mmol/L					P-value
	2.3–3.4	3.5–3.9	4.0–4.4	4.5–4.9	5.0–6.3	
Subjects, %	24 (0.5)	404 (7.9)	2,873 (56.0)	1,636 (31.9)	193 (3.8)	<0.001
Women, %	20 (83.3)	279 (69.1)	1588 (55.3)	790 (48.3)	90 (46.6)	<0.001
Age, y	53.4 ± 12.5	48.6 ± 12.5	48.1 ± 11.7	48.4 ± 11.7	48.6 ± 11.9	0.20
Height, cm	166 ± 9	171 ± 9	173 ± 9	174 ± 10	174 ± 9	<0.001
Weight, kg	73 ± 15	75 ± 14	77 ± 13	77 ± 13	77 ± 14	0.01
BMI, kg/m^2^	26.4 ± 4.5	25.7 ± 4.3	25.7 ± 3.9	25.6 ± 3.9	25.4 ± 3.8	0.53
Current smoker, %	6 (25.0)	74 (18.3)	826 (28.8)	627 (38.3)	68 (35.2)	<0.001
No alcohol consumption, %	12 (50.0)	114 (28.2)	681 (23.7)	361 (22.1)	34 (17.6)	<0.001
White, %	20 (83.3)	371 (92.5)	2,736 (95.9)	1,575 (97.0)	189 (97.9)	<0.001
Education, high, %	2 (8.3)	126 (31.2)	965 (33.6)	520 (31.8)	64 (33.2)	0.07
Diabetes Mellitus, %	4 (16.7)	10 (2.5)	35 (1.2)	34 (2.1)	3 (1.6)	<0.001
Family history of CKD, %	0 (0)	3 (0.7)	41 (1.4)	22 (1.3)	5 (2.6)	0.45
Systolic blood pressure, mm Hg	134 ± 19	127 ± 20	125 ± 18	125 ± 18	125 ± 18	0.03
Diastolic blood pressure, mm Hg	77 ± 10	73 ± 10	72 ± 9	73 ± 9	72 ± 8	0.15
Cardiovascular disease, %	1 (4.2)	11 (2.7)	85 (3.0)	64 (3.9)	9 (4.7)	0.34
Medication:						
ACEi*, %	1 (5.0)	18 (5.0)	78 (3.2)	37 (2.7)	4 (2.6)	0.23
ARB*, %	0 (0)	2 (0.6)	9 (0.4)	8 (0.6)	1 (0.6)	0.89
Beta blockers*, %	5 (25.0)	29 (8.1)	135 (5.5)	81 (5.8)	11 (7.1)	0.002
Thiazide diuretics*, %	6 (30.0)	35 (9.7)	43 (1.8)	8 (0.6)	1 (0.6)	<0.001
Loop diuretics*, %	1 (5.0)	6 (1.7)	8 (0.3)	7 (0.5)	1 (0.6)	0.001
Potassium-sparing diuretics*, %	0 (0)	17 (4.7)	17 (0.7)	4 (0.3)	4 (2.6)	<0.001
Proton pump inhibitors*, %	0 (0)	12 (3.0)	63 (2.3)	51 (3.2)	7 (3.7)	0.28
Lipid lowering drugs, %	1 (4.2)	28 (6.9)	136 (4.7)	101 (6.2)	11 (5.7)	0.17
Glucose lowering drugs, %	0 (0)	5 (1.2)	20 (0.7)	26 (1.6)	1 (0.5)	0.06
Plasma potassium, mmol/L	3.2 ± 0.3	3.8 ± 0.1	4.2 ± 0.1	4.6 ± 0.1	5.1 ± 0.2	<0.001
Plasma magnesium, mmol/L	0.80 ± 0.07	0.80 ± 0.06	0.81 ± 0.06	0.82 ± 0.05	0.83 ± 0.05	0.30
Plasma albumin, g/L	44.8 ± 2.9	45.3 ± 2.9	45.9 ± 2.6	45.9 ± 2.6	46.6 ± 2.5	<0.001
Plasma sodium, mmol/L	141 ± 4	141 ± 3	142 ± 2	142 ± 2	142 ± 3	0.18
Plasma chloride, mmol/L	101 ± 5	105 ± 8	106 ± 2	106 ± 2	106 ± 2	<0.001
Plasma aldosterone†, ng/dL	13.1 ± 8.0	14.2 ± 7.8	13.3 ± 6.9	12.9 ± 6.6	12.4 ± 5.1	0.12
Plasma PTH‡, pmol/L	5.6 ± 2.1	4.0 ± 1.4	3.8 ± 1.4	3.8 ± 1.5	3.8 ± 1.7	<0.001
Plasma 1,25 dihydroxyvitamin D§, pg/mL	57.4 ± 23.8	58.1 ± 20.0	55.8 ± 18.2	55.3 ± 17.7	55.2 ± 17.8	0.10
Plasma phosphorus, mg/dL	3.15 ± 0.55	3.10 ± 0.50	3.11 ± 0.48	3.16 ± 0.49	3.22 ± 0.51	0.001
Plasma glucose, mmol/L	5.4 ± 2.9	4.8 ± 1.3	4.7 ± 0.8	4.8 ± 0.9	4.7 ± 0.7	0.89
hs-CRP¶, mg/L	3.3 ± 3.4	2.8 ± 5.5	2.2 ± 4.0	2.2 ± 3.2	2.0 ± 2.7	0.01
BUN, mg/dL	13.8 ± 4.9	13.8 ± 3.6	14.4 ± 3.3	14.8 ± 3.5	14.9 ± 3.4	<0.001
Serum creatinine, mg/dL	0.7 (0.6–0.80)	0.7 (0.6–0.8)	0.8 (0.7–0.9)	0.8 (0.7–0.9)	0.8 (0.7–0.9)	<0.001
BUN/creatinine ratio	17.8 (13.6–22.0)	17.9 (15.1–21.6)	18.0 (15.3–21.3)	17.7 (15.1–21.3)	17.6 (14.8–21.4)	0.82
eGFR, ml/min/1.73 m^2^	91 ± 21	99 ± 15	98 ± 15	96 ± 15	95 ± 15	0.06
Urinary excretion of:						
Potassium, mmol/24h	60.3 (41.3–70.3)	62.9 (48.8–80.6)	70.3 (56.9–83.9)	70.6 (58.2–85.3)	72.6 (60.2–88.8)	<0.001
Sodium, mmol/24h	98 (80–148)	125 (98–148)	135 (104–169)	136 (107–167)	131 (106–169)	<0.001
Magnesium, mmol/24h	2.54 (1.31–3.80)	3.58 (2.64–4.43)	3.76 (2.89–4.72)	3.88 (2.99–4.85)	3.78 (3.04–5.06)	<0.001
Urea, mmol/24h	288 (183–388)	324 (259–389)	343 (284–413)	348 (284–419)	337 (277–423)	<0.001
Creatinine, mmol/24	9.2 (7.3–11.7)	10.8 (9.2–13.1)	11.7 (9.5–14.3)	11.9 (9.8–14.7)	11.4 (9.5–14.4)	<0.001
Albumin, mg/24h	8.3 (7.3–21.3)	8.2 (6.1–11.4)	7.8 (5.8–11.0)	7.6 (5.7–11.1)	8.0 (5.8–11.6)	0.02

Continuous variables are reported as mean ± SD or median (interquartile range) and categorical variables are reported as *n* (%).

* Data available in 4,378 subjects.

^†^ Data available in 5,008 subjects.

^§^ Data available in 4,971 subjects.

^‡^ Data available in 4,967 subjects.

^¶^ Data available in 4,959 subjects.

Abbreviations: ACEi, angiotensin converting enzyme inhibitor; ARB, angiotensin receptor blockers; BMI, body mass index; BUN, blood urea nitrogen; hs-CRP, high-sensitivity C-reactive protein; PTH, parathyroid hormone.

Both eGFR and UAE were regularly measured at baseline and at several follow-up screening waves, of which the first took place at a median follow-up of 4.1 years (IQR, 4.0–4.3 years), the second at 6.3 years (IQR, 6.0–6.7 years), the third at 9.2 years (IQR, 8.8–9.5 years), and the fourth at 11.5 years (IQR, 11.2–12.4 years).

### Risk factors for hypokalemia and hyperkalemia

Univariable ORs and 95% CIs according to categories of plasma potassium are shown in S1 Table. Bivariable ORs (95% CIs) in which associations were adjusted for the potential influence of eGFR are shown in Table 2. Female sex, alcohol consumption, diabetes, hypertension, use of beta blockers, use of thiazide and loop diuretics, and UAE were associated with higher risk of hypokalemia, whereas high educational level, and higher urinary potassium and magnesium excretion were associated with lower risk of hypokalemia, after accounting for eGFR. Female sex and alcohol consumption were associated with a lower risk of hyperkalemia. Use of potassium-sparing diuretics, urinary excretions of potassium and magnesium were associated with higher risk of hyperkalemia, independent of eGFR.

**Table 2:** Bivariable adjusted odds ratios (95% confidence intervals) combined with eGFR for risk factors of hypokalemia and hyperkalemia in 5,130 participants of the Prevention of Renal and Vascular End-Stage Disease (PREVEND) study.

	Plasma potassium, mmol/L				
	2.3–3.4	3.5–3.9	4.0–4.4	4.5–4.9	5.0–6.3
Sex, female vs male	4.00 (1.36–11.72)*	1.81 (1.45–2.26)***	1.00 (ref)	0.75 (0.67–0.85)***	0.70 (0.53–0.94)*
Age, y	1.02 (0.98–1.07)	1.01 (1.00–1.03)*	1.00 (ref)	0.99 (0.99–1.00)*	0.99 (0.97–1.00)
BMI, kg/m^2^	1.02 (0.92–1.13)	1.00 (0.98–1.03)	1.00 (ref)	0.99 (0.97–1.00)	0.97 (0.93–1.01)
Current smoker, yes vs no	0.82 (0.33–2.08)	0.56 (0.43–0.73)***	1.00 (ref)	1.53 (1.35–1.74)***	1.34 (0.99–1.82)
Alcohol consumption, yes vs no	2.96 (1.32–6.67)**	1.29 (1.02–1.62)*	1.00 (ref)	0.89 (0.77–1.03)	0.66 (0.45–0.96)*
Education, high, yes vs no	0.18 (0.04–0.77)*	0.90 (0.72–1.12)	1.00 (ref)	0.92 (0.81–1.05)	0.98 (0.72–1.34)
White, yes vs no	0.18 (0.02–1.40)	0.66 (0.29–1.51)	1.00 (ref)	2.68 (1.11–6.45)*	-
Type 2 Diabetes, yes vs no	13.88 (4.44–43.35)***	2.13 (1.04–4.34)*	1.00 (ref)	1.63 (1.01–2.63)*	1.18 (0.36–3.87)
Hypertension, yes vs no	6.74 (2.54–17.92)***	1.62 (1.29–2.05)***	1.00 (ref)	0.81 (0.70–0.94)**	0.75 (0.52–1.07)
ACEi, yes vs no	1.20 (0.16–9.28)	1.71 (1.01–2.90)	1.00 (ref)	0.76 (0.51–1.13)	0.69 (0.25–1.92)
ARB, yes vs no	-	1.64 (0.35–7.66)	1.00 (ref)	1.40 (0.54–3.65)	1.45 (0.18–11.61)
Beta blockers, yes vs no	4.61 (1.59–13.39)**	1.61 (1.06–2.47)*	1.00 (ref)	0.97 (0.73–1.29)	1.13 (0.59–2.16)
Thiazide diuretics, yes vs no	20.12 (7.05–57.40)***	6.65 (4.16–10.63)***	1.00 (ref)	0.29 (0.14–0.63)**	0.31 (0.04–2.26)
Loop diuretics, yes vs no	10.94 (1.25–95.73)*	5.71 (1.96–16.66)**	1.00 (ref)	1.36 (0.49–3.77)	1.60 (0.20–12.95)
Potassium-sparing diuretics, yes vs no	-	8.00 (4.01–15.96)***	1.00 (ref)	0.37 (0.12–1.09)	3.14 (1.04–9.55)*
Urinary potassium excretion, mmol/24h	0.96 (0.94–0.98)**	0.99 (0.98–0.99)***	1.00 (ref)	1.00 (1.00–1.01)*	1.01 (1.00–1.01)*
Urinary magnesium excretion, mmol/24h	0.55 (0.41–0.74)***	0.89 (0.83–0.95)**	1.00 (ref)	1.05 (1.01–1.09)*	1.14 (1.04–1.25)**
Urinary albumin excretion, mg/24h†	3.00 (1.39–6.48)**	1.15 (0.92–1.43)	1.00 (ref)	0.97 (0.85–1.10)	1.14 (0.84–1.54)

ORs (95% CIs) are calculated with multinomial regression analyses.

* P<0.05,

**P<0.01,

***P<0.001.

^†^ Natural log(ln)-transformed.

Abbreviations: ACEi, angiotensin converting enzyme inhibitor; ARB, angiotensin receptor blockers; BMI, body mass index; eGFR, estimated glomerular filtration rate.

### Risk of developing CKD

During a median follow-up of 10.3 years (IQR: 6.3–11.4 years), 753 subjects developed CKD. In the crude model, subjects with hypokalemia had a higher risk of developing CKD compared to subjects with normokalemia (HR, 5.31; 95% CI, 2.99–9.24; Table 3). After adjustment of age, sex, eGFR, height, weight, urinary potassium excretion, and use of diuretics, this association remained significant (HR, 3.65; 95% CI, 1.94–6.85). Further adjustment for systolic blood pressure, plasma aldosterone, plasma albumin, plasma magnesium, hs-CRP, type 2 diabetes, smoking, alcohol, education, race, urinary creatinine excretion, plasma chloride, or the BUN/creatinine ratio did not materially alter the association. Secondary analyses in which CKD was defined as either eGFR <60 ml/min/1.73 m^2^ or UAE >30 mg/24h alone, rendered essentially similar results (S2 Table).

**Table 3:** Association of plasma potassium with risk of developing chronic kidney disease defined as eGFR_creatinine-cystatin C_ <60 ml/min/1.73m^2^ or urinary albumin excretion >30 mg/24h in 5,130 participants of the Prevention of Renal and Vascular End-Stage Disease (PREVEND) study.

	Plasma potassium, mmol/L				
	2.3–3.4	3.5–3.9	4.0–4.4	4.5–4.9	5.0–6.3
Person-years	149	3541	25,767	14,434	1,644
Number of events	12	63	401	249	28
Rates*	805	178	156	173	170
Crude model	5.31 (2.99–9.24)	1.14 (0.88–1.49)	1.00 (ref)	1.11 (0.95–1.30)	1.09 (0.75–1.61)
Multivariable model 1†	4.33 (2.43–7.72)	1.18 (0.91–1.55)	1.00 (ref)	1.02 (0.87–1.20)	1.00 (0.69–1.47)
Multivariable model 2‡	3.65 (1.94–6.85)	0.83–0.61–1.14)	1.00 (ref)	1.07 (0.90–1.26)	1.00 (0.66–1.53)
+ Systolic blood pressure	3.70 (1.97–6.93)	0.81 (0.60–1.11)	1.00 (ref)	1.08 (0.91–1.28)	1.04 (0.68–1.59)
+ Aldosterone	3.11 (1.60–6.04)	0.88 (0.64–1.21)	1.00 (ref)	1.04 (0.86–1.24)	1.08 (0.70–1.69)
+ Plasma albumin	3.65 (1.94–6.86)	0.83 (0.61–1.14)	1.00 (ref)	1.07 (0.90–1.27)	1.00 (0.65–1.52)
+ Plasma magnesium	3.36 (1.78–6.33)	0.82 (0.60–1.12)	1.00 (ref)	1.05 (0.89–1.25)	1.01 (0.66–1.55)
+ Hs-CRP	3.94 (2.09–7.41)	0.86 (0.63–1.18)	1.00 (ref)	1.09 (0.92–1.30)	1.03 (0.67–1.58)
+ Type 2 diabetes	3.27 (1.75–6.11)	0.83 (0.61–1.14)	1.00 (ref)	1.05 (0.89–1.24)	1.02 (0.67–1.56)
+ Smoking	3.65 (1.94–6.86)	0.84 (0.62–1.15)	1.00 (ref)	1.06 (0.89–1.26)	1.00 (0.65–1.52)
+ Alcohol consumption	3.40 (1.81–6.41)	0.83 (0.60–1.13)	1.00 (ref)	1.07 (0.90–1.27)	1.01 (0.66–1.54)
+ Education	3.61 (1.92–6.78)	0.83 (0.61–1.13)	1.00 (ref)	1.07 (0.90–1.26)	1.00 (0.66–1.53)
+ Race	3.70 (1.97–6.95)	0.83 (0.61–1.14)	1.00 (ref)	1.07 (0.90–1.27)	1.01 (0.66–1.55)
+ Urinary creatinine excretion	3.70 (1.97–6.95)	0.82 (0.60–1.12)	1.00 (ref)	1.07 (0.90–1.26)	1.01 (0.66–1.54)
+ Plasma chloride	3.50 (1.83–6.71)	0.83 (0.61–1.13)	1.00 (ref)	1.07 (0.90–1.27)	1.00 (0.66–1.53)
+ BUN/creatinine ratio	3.66 (1.95–6.88)	0.84 (0.61–1.14)	1.00 (ref)	1.06 (0.90–1.26)	1.00 (0.66–1.53)

Hazard ratios (HR) and 95% confidence intervals were derived from Cox proportional hazards regression models.

* Number of events divided by time at risk standardized per 10,000 person-years.

^†^ Multivariable model 1 is adjusted for age, sex, and eGFR.

^‡^ Multivariable model 2 is additionally adjusted for height, weight, urinary potassium excretion, and use of diuretics.

Abbreviations: BUN, blood urea nitrogen; eGFR, estimated glomerular filtration rate; hs-CRP, high-sensitivity C-reactive protein.

We found neither an association of hyperkalemia with risk of developing CKD in the crude model (HR, 1.09; 95% CI, 0.75–1.61; Table 3), nor after multivariable adjustment for age, sex, eGFR, height, weight, urinary potassium excretion, and use of diuretics (HR, 1.00; 95% CI, 0.66–1.53).

### Effect modification by diuretic use

**Fig 2:**
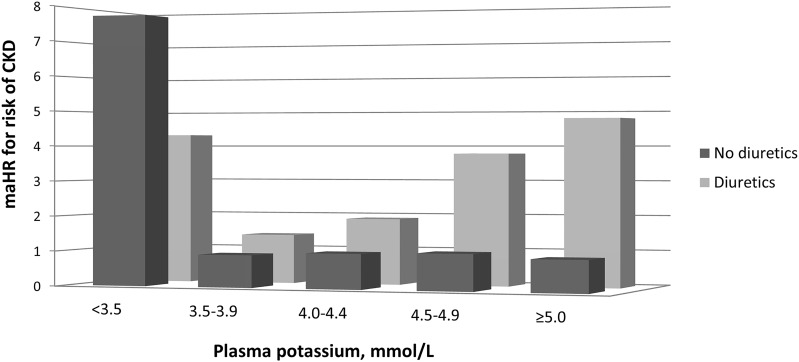
Association of plasma potassium with risk of developing chronic kidney disease stratified by use of diuretics in 5,130 participants of the Prevention of Renal and Vascular End-Stage Disease (PREVEND) study. Hazard ratios were calculated with Cox proportional hazards regression models and were adjusted for age, sex, height, weight, eGFR, and urinary potassium excretion. The category plasma potassium 4.0–4.4 mmol/L and no diuretics was set as reference. Abbreviations: CKD, chronic kidney disease; eGFR, estimated glomerular filtration rate; maHR, multivariable-adjusted hazard ratio.

The association of plasma potassium with risk of developing CKD was modified by use of diuretics (P_interaction_ = 0.02; Fig 2). Hypokalemia was associated with a higher risk of developing CKD in both subjects using diuretics (HR, 4.32; 95% CI, 1.77–10.51) and not using diuretics (HR, 7.74; 95% CI, 3.43–17.48), compared to subjects with normokalemia and not using diuretics (S3 Table). Moreover, in the absence of hypokalemia, plasma potassium was associated with an increased risk of CKD in subjects using diuretics (P_trend_ = 0.01) but not in subjects not using diuretics (P_trend_ = 0.74). We found no further evidence for effect modification by sex, age, hypertension status, urinary potassium excretion, baseline eGFR, and plasma aldosterone (P_interaction_>0.1).

### Sensitivity analyses for risk of CKD

When stratifying the analyses by use of potassium-wasting diuretics, hypokalemia was associated with a higher risk of developing CKD in both subjects using potassium-wasting diuretics (HR, 4.24; 95% CI, 1.74–10.32) and not using potassium-wasting diuretics (HR, 7.58; 95% CI, 3.36–17.10), compared to subjects with normokalemia not using potassium-wasting diuretics. Since there were no subjects using potassium-sparing diuretics among the hypokalemic subjects, we could not examine the specific role of potassium-sparing diuretics in the association of hypokalemia with risk of CKD. Second, results were essentially the same when we excluded subjects at baseline with an eGFR <66 (instead of <60) ml/min/1.73m^2^ and/or a UAE <25 (instead of <30) mg/24h (N = 4,923, n = 539) for a more pronounced decline in kidney function during follow-up before reaching the CKD endpoint (HR for hypokalemia compared to normokalemia, 3.62; 95% CI, 1.80–7.30). Third, when we restricted the analyses to subjects who were not using antihypertensive drugs at baseline (N = 4,507, n = 438) results for risk of CKD did not materially change (HR for hypokalemia compared to normokalemia, 6.30; 95% CI, 1.55–25.65). Fourth, we excluded subjects from the analysis who developed diabetes during follow-up (N = 423). During a median follow-up of 10.4 years (IQR: 6.4–11.4 years), 541 subjects developed CKD. The results of this sensitivity analysis did not differ materially from that of the primary analyses, with hypokalemia associated with a higher risk of developing CKD in both subjects using diuretics (HR, 3.81; 95% CI, 1.41–10.30) and not using diuretics (HR, 8.43; 95% CI, 3.11–22.88), compared to subjects with normokalemia and not using diuretics. Finally, in weighted analyses that accounted for the sampling design of the study, results were slightly attenuated (HR for hypokalemia compared to normokalemia, 2.98; 95% CI, 0.79–11.31). However, when stratified for use of diuretics, hypokalemic subjects not using diuretics had a significant increased risk of developing CKD (HR, 8.72; 95% CI, 3.66–20.81, compared to subjects with normokalemia not using diuretics).

## Discussion

In this prospective population-based cohort study, hypokalemia was associated with a higher risk of developing CKD, regardless of diuretic use. The association between plasma potassium and risk of CKD was modified by use of diuretics in such a way that non-hypokalemic potassium concentrations were associated with an increased CKD risk among subjects using diuretics but not among subjects not using diuretics. The associations remained after adjustment of confounders, CKD risk factors, and potential mediators of the association, such as systolic blood pressure, plasma magnesium, plasma albumin, hs-CRP, aldosterone, plasma chloride, and the BUN/creatinine ratio.

An observational study in healthy Japanese subjects not using diuretics observed that subjects with lower plasma potassium concentrations had an increased risk of developing CKD (potassium <4.0 mmol/L; HR, 2.65; 95% CI, 2.04–3.44) [13]. These results are in line with a recent study of Chen et al., who investigated the association of potassium concentrations and risk of CKD in the Atherosclerosis Risk of Community Study, a general population cohort from the United States [14]. They observed that hypokalemia was associated with adverse kidney outcomes among participants not taking potassium-wasting drugs, i.e. loop diuretics, thiazide diuretics, and thiazide-like diuretics (potassium <3.5 mmol/24h; HR 1.65; 95 CI, 1.16–2.35). In our present study, we observed that hypokalemia was associated with a higher risk of developing CKD, regardless of diuretic use. Moreover, in contrast to Chen et al., among subjects using diuretics, non-hypokalemic plasma potassium concentrations were positively associated with risk of CKD. The latter finding might reflect confounding by indication or an adverse effect of diuretics.

The prevalence of hypokalemia at baseline in the Prevention of Renal and Vascular End-stage Disease (PREVEND) study (0.5%) was lower compared to a previous reported prevalence of hypokalemia (also defined as potassium <3.5 mmol/L) in the third National Health and Nutrition Examination Survey (NHANES III) study (3.1%), a general population cohort in the US [24]. The prevalence of hyperkalemia (defined as ≥5.0 mmol/L) was higher in the PREVEND study (3.8%) compared to the NHANES III study (0.7%). These difference in prevalences of hypo- and hyperkalemia might be explained by the lower number of Black subjects included in our cohort (0.9%) compared to the NHANES III (10.4%) [25], since Black people are known to have lower potassium concentrations compared to White people [26].

Our data regarding the determinants of potassium abnormalities mainly confirmed previous observations. For example, previous studies also identified sex as a risk factor for hypo- and hyperkalemia, where women had higher risk of hypokalemia and men had higher risk of hyperkalemia [7,11,24,27,28], possibly because of differences in body composition. Diuretic use is also known in the literature as a risk factor for hypokalemia [4,7,11,29], due to increased renal potassium loss. Renal insufficiency is reported to be the most common cause of hyperkalemia [30]. In the present study, these risk factors were all associated with hyperkalemia independent of eGFR. No associations of hypokalemia and hyperkalemia with age were observed in our study, while other studies argue that increasing age is an important risk factor for both conditions, especially hyperkalemia [27,28,31].

Several possible mechanisms by which hypokalemia could induce kidney injury are proposed in literature. We investigated some of these possible underlying mechanisms in the present study. First, despite the strict regulation of plasma potassium concentrations between narrow limits and the subsequent limited effect of potassium intake on plasma potassium concentrations [32–37], in some cases hypokalemia can be a reflection of insufficient potassium intake–and thereby also lower intake of fiber, vitamins and antioxidants–or poor nutritional status, which are both associated with risk of developing CKD via oxidative stress, hypertension, or inflammation [38–41]. Despite that subjects with hypokalemia had a significantly lower potassium excretion in the present study, when additionally adjusting for urinary potassium excretion, results remained similar for the association of hypokalemia with risk of CKD. Second, high plasma potassium is associated with reduced activity of the renin-angiotensin-aldosterone system, which has been shown to slow progression of renal disease [42], whereas hypokalemia was reported to stimulate renin and angiotensin II despite direct suppression of aldosterone synthesis leading to salt-sensitive hypertension, intrarenal vasoconstriction and ischemia [10,43–45]. Hypokalemic subjects in the present study had a higher blood pressure and were more likely to use antihypertensive medication, however, results for risk of CKD were essentially the same when additionally adjusting for systolic blood pressure or aldosterone. Third, experimental studies determined that induced hypokalemia led to declines in insulin release in response to hyperglycemia and to a decrease in pancreatic β-cell sensitivity to hyperglycemia with a reduction in insulin release [46–48]. Although subjects with hypokalemia were more likely to have diabetes, additional adjustment for diabetes or excluding subjects who developed diabetes during follow-up rendered similar results as the primary analyses. Finally, low potassium concentrations could have induced diabetes insipidus or could have been accompanied with metabolic alkalosis. However, additional adjustment for the BUN/creatinine ratio or serum chloride levels did not alter the association of hypokalemia with risk of CKD. Taken together, these results suggest that other mechanisms are responsible for the observed association between hypokalemia and risk of CKD. Other mechanisms proposed in literature involves hypokalemia-induced tubulointerstitial injury by ammoniagenesis which has been observed in experimental animal models [9,49]. In line with these studies, our results show that hypokalemia is associated with increased UAE, which can be considered a proxy of tubulointerstitial damage [50,51]. However, we do not have data on urinary ammonia excretion, so we could not further investigate this mechanism. Another mechanism proposed includes the renoprotective effects of higher plasma potassium by upregulating renal kinins, such as kallikrein [52]. Experimental studies showed that an increase in plasma potassium concentration reduces cultured vascular smooth muscle cell proliferation [53], inhibits free radical formation from endothelial cells and macrophages [54], and inhibits platelet aggregation and arterial thrombosis [55,56]. However, also no data on plasma kallikrein were available in the PREVEND study, so we could not investigate this possible mechanism.

The absence of an association between hyperkalemia and risk of developing CKD in the present study is consistent with the findings of Chen *et al*. and Fukui *et al*. [13,14]. Both studies did not find associations of high plasma potassium concentrations with risk of developing CKD in subjects free of CKD at baseline and not taking (potassium-sparing) diuretics. Interestingly, Chen and colleagues also observed that higher potassium concentrations were associated with CKD in participants taking potassium-wasting diuretics[14]. Furthermore, studies in CKD patients did not observe associations of hyperkalemia with risk of ESRD [6,12], expect for one study [11]. Moreover, It is important to realize that due to the observational design of our study, and that of Chen *et al*. and Fukui *et al*., conclusions about no causal relationship between hyperkalemia and risk of CKD cannot be drawn.

We observed in our study that both the use of diuretics and beta blockers were associated with hypokalemia in cross-sectional analyses, which raises several interesting insights. Diuretics are often used in patients with heart and/or renal disease with volume overload and are associated with potassium loss via the urine. Further, experimental studies as well as clinical and observational studies have suggested a correlation between diuretic use and progression of renal injury [57]. Thiazides have been proposed to associate with renal injury via the induction of hypokalemia, metabolic abnormalities, and volume depletion and to lead to additional focal glomerular damage not observed in states of equivalent hypokalemia in the absence of diuretics [58].

Some limitations of this study should be mentioned. First, as with any observational study, there may be unmeasured or residual confounding despite the substantial number of potentially confounding factors for which we adjusted. Second, due to the small number of subjects with hypo- and hyperkalemia, we could not calculate ORs (95% CIs) for use of thiazide and potassium-sparing diuretics and race for all potassium categories, or reach significance due to the large 95% CIs. However, despite the small number of subjects in some of the potassium categories, we observed a significant association of hypokalemia with increased risk of CKD. Unfortunately, the underlying cause of hypokalemia could not be identified. Third, our results may not be readily generalizable to different demographic populations because >95% of the individuals of the PREVEND study are white. Fourth, our results may not be readily generalizable to different demographic populations because >95% of the individuals of the PREVEND study are White. Blacks, for instance, typically show a lower urinary excretion rate of potassium than do whites [59,60] due to a higher loss of potassium via other routes, a slower rate of disposal as a result of slower skeletal muscle uptake of potassium, or genetic differences in renal potassium handling [60].

Finally, plasma potassium was measured only at baseline. However, when the intra-individual variability of variables is taken into account, this results in strengthening of associations [61]. Therefore, our use of a single plasma potassium measurement at baseline rather than several or multiple ones, will likely provide an underestimation of the true effect.

A strength of this study is the use of the combined CKD end point consisting of a creatinine-cystatin C-based eGFR and two consecutive 24-hour UAE at each screening to ascertain CKD events. Other strengths of this study were the prospective design, the relatively large sample size, and the availability of detailed data on potential confounders.

In conclusion, in this Dutch prospective population-based study, hypokalemia was associated with an increased risk of developing CKD, regardless of diuretic use. Furthermore, the association between plasma potassium and risk of CKD was modified by use of diuretics, in such a way that in the absence of hypokalemia, plasma potassium was associated with an increased risk of CKD in subjects using diuretics, but not in subjects not using diuretics. Traditionally, the concern for nephrologists is hyperkalemia. However, these data show that hypokalemia might be a risk factor for developing CKD among subjects with normal kidney function. Further research is needed to identify the role of the underlying cause of hypokalemia in this association, and to investigate whether the higher risk of developing CKD could be diminished when regulating plasma potassium levels strictly.

## Supporting information

S1 Table Univariable odds ratios (95% confidence intervals) for risk factors of hypokalemia and hyperkalemia in 5,130 participants of the Prevention of Renal and Vascular End-Stage Disease (PREVEND) study. Odds ratios (95% confidence intervals) are calculated with multinomial regression analyses. * P<0.05, ** P<0.01, *** P<0.001. † Natural log(ln)-transformed. Abbreviations: ACEi, angiotensin converting enzyme inhibitor; ARB, angiotensin receptor blockers; BMI, body mass index; PREVEND, Prevention of Renal and Vascular End-Stage Disease.
(DOCX)

S2 Table Association of plasma potassium with risk of developing chronic kidney disease defined as development of either eGFR <60 ml/min/1.73 m^2^ or UAE >30 mg/24h alone in 5,130 participants of the Prevention of Renal and Vascular End-Stage Disease (PREVEND) study. Hazard ratios and 95% confidence intervals were derived from Cox proportional hazards regression models. * Number of events divided by time at risk standardized per 10,000 person-years. † Multivariable model 1 is adjusted for age, sex, and eGFR. ‡ Multivariable model 2 is additionally adjusted for height, weight, urinary potassium excretion, and use of diuretics. Abbreviations: eGFR, estimated glomerular filtration rate; hs-CRP, high-sensitivity C-reactive protein; PREVEND, Prevention of Renal and Vascular End-Stage Disease; UAE, urinary albumin excretion.
(DOCX)

S3 Table Association of plasma potassium with risk of developing chronic kidney disease stratified by use of diuretics in 5,130 participants of the Prevention of Renal and Vascular End-Stage Disease (PREVEND) study. Hazard ratios and 95% confidence intervals were derived from Cox proportional hazards regression models. * Number of events divided by time at risk standardized per 10,000 person-years. † Multivariable model 1 is adjusted for age, sex, eGFR, height, weight, and urinary potassium excretion. Abbreviations: eGFR, estimated glomerular filtration rate; PREVEND, Prevention of Renal and Vascular End-Stage Disease.
(DOCX)
