# Supplementary material for: Plasma potassium, diuretic use and risk of developing chronic kidney disease in a predominantly White population
Source: PLoS One. 2017 Mar 27;12(3):e0174686. doi: 10.1371/journal.pone.0174686 (PMC5367826; doi:10.1371/journal.pone.0174686)
Supplement: S1 Table — Odds ratios (95% confidence intervals) are calculated with multinomial regression analyses. * P<0.05, ** P<0.01, *** P<0.001. † Natural log(ln)-transformed. Abbreviations: ACEi, angiotensin converting enzyme inhibitor; ARB, angiotensin receptor blockers; BMI, body mass index; PREVEND, Prevention of Renal and Vascular End-Stage Disease. (DOCX) [file pone.0174686.s001.docx]

**S1 Table.** Univariable odds ratios (95% confidence intervals) for risk factors of hypokalemia and hyperkalemia in 5,130 participants of the Prevention of Renal and Vascular End-stage Disease (PREVEND) study.

|  | **Plasma potassium, mmol/L** | | | | |
| --- | --- | --- | --- | --- | --- |
|  | 2.3-3.4 | 3.5-3.9 | 4.0-4.4 | 4.5-4.9 | 5.0-6.3 |
| Sex, female vs male | 4.05 (1.38-11.87)* | 1.81 (1.45-2.26)*** | 1.00 (ref) | 0.76 (0.67-0.85)*** | 0.71 (0.53-0.95)* |
| Age, y | 1.04 (1.00-1.07)* | 1.00 (1.00-1.01) | 1.00 (ref) | 1.00 (1.00-1.01) | 1.00 (0.99-1.02) |
| BMI, kg/m^2^ | 1.04 (0.95-1.14) | 1.00 (0.97-1.03) | 1.00 (ref) | 0.99 (0.98-1.01) | 0.98 (0.94-1.02) |
| eGFR, ml/min/1.73m^2^ | 0.97 (0.95-1.00)* | 1.01 (1.00-1.01) | 1.00 (ref) | 0.99 (0.99-1.00)*** | 0.99 (0.98-1.00)** |
| Current smoker, yes vs no | 0.83 (0.33-2.09) | 0.56 (0.43-0.72)*** | 1.00 (ref) | 1.54 (1.36-1.75)*** | 1.35 (0.99-1.83) |
| Alcohol consumption, yes vs no | 3.22 (1.44-7.20)** | 1.27 (1.00-1.60)* | 1.00 (ref) | 0.91 (0.79-1.05) | 0.69 (0.47-1.01) |
| Education, high, yes vs no | 0.20 (0.05-0.86)* | 0.88 (0.70-1.10) | 1.00 (ref) | 0.96 (0.84-1.09) | 1.04 (0.76-1.42) |
| White, yes vs no | 0.25 (0.03-1.92) | 0.62 (0.27-1.41) | 1.00 (ref) | 2.96 (1.23-7.12)* | - |
| Type 2 diabetes, yes vs no | 16.22 (5.27-49.91)*** | 2.06 (1.0-4.19)* | 1.00 (ref) | 1.72 (1.07-2.77)* | 1.28 (0.39-4.20) |
| Hypertension, yes vs no | 7.96 (3.13-20.26)*** | 1.49 (1.19-1.86)** | 1.00 (ref) | 0.91 (0.79-1.04) | 0.87 (0.62-1.23) |
| ACEi, yes vs no | 1.60 (0.21-12.10) | 1.60 (0.95-2.70) | 1.00 (ref) | 0.83 (0.56-1.23) | 0.80 (0.29-2.21) |
| ARB, yes vs no | - | 1.51 (0.33-7.04) | 1.00 (ref) | 1.56 (0.60-4.06) | 1.75 (0.22-13.89) |
| Beta blockers, yes vs no | 5.72 (2.05-15.96)*** | 1.50 (0.99-2.28) | 1.00 (ref) | 1.06 (0.80-1.41) | 1.30 (0.69-2.46) |
| Thiazide diuretics, yes vs no | 23.98 (8.80-65.37)*** | 6.03 (3.80-9.56)*** | 1.00 (ref) | 0.32 (0.15-0.70)** | 0.36 (0.05-2.64) |
| Loop diuretics, yes vs no | 16.05 (1.91-134.72)* | 5.17 (1.78-14.99)** | 1.00 (ref) | 1.54 (0.56-4.25) | 1.97 (0.25-15.83) |
| Potassium-sparing diuretics, yes vs no | - | 7.09 (3.59-14.02)*** | 1.00 (ref) | 0.41 (0.14-1.23) | 3.77 (1.25-11.33)* |
| Urinary potassium excretion, mmol/24h | 0.96 (0.94-0.98)*** | 0.99 (0.98-1.00)*** | 1.00 (ref) | 1.00 (1.00-1.01) | 1.01 (1.00-1.01) |
| Urinary magnesium excretion, mmol/24h | 0.55 (0.41-0.75)*** | 0.89 (0.83-0.95)** | 1.00 (ref) | 1.05 (1.01-1.09)* | 1.13 (1.03-1.24)** |
| Urinary albumin excretion, mg/24h† | 3.23 (1.48-7.04)** | 1.14 (0.92-1.42) | 1.00 (ref) | 0.98 (0.86-1.11) | 1.15 (0.85-1.56) |

Odds ratios (95% confidence intervals) are calculated with multinomial regression analyses.

* P<0.05, ** P<0.01, *** P<0.001.

† Natural log(ln)-transformed.

Abbreviations: ACEi, angiotensin converting enzyme inhibitor; ARB, angiotensin receptor blockers; BMI, body mass index; PREVEND, Prevention of Renal and Vascular End-Stage Disease.
