# Supplementary material for: Plasma potassium, diuretic use and risk of developing chronic kidney disease in a predominantly White population
Source: PLoS One. 2017 Mar 27;12(3):e0174686. doi: 10.1371/journal.pone.0174686 (PMC5367826; doi:10.1371/journal.pone.0174686)
Supplement: S2 Table — Hazard ratios and 95% confidence intervals were derived from Cox proportional hazards regression models. * Number of events divided by time at risk standardized per 10,000 person-years. † Multivariable model 1 is adjusted for age, sex, and eGFR. ‡ Multivariable model 2 is additionally adjusted for height, weight, urinary potassium excretion, and use of diuretics. Abbreviations: eGFR, estimated glomerular filtration rate; hs-CRP, high-sensitivity C-reactive protein; PREVEND, Prevention of Renal and Vascular End-Stage Disease; UAE, urinary albumin excretion. (DOCX) [file pone.0174686.s002.docx]

**S2 Table**. Association of plasma potassium with risk of developing chronic kidney disease defined as development of either eGFR <60 ml/min/1.73 m^2^ or UAE >30 mg/24h alone in 5,130 participants of the Prevention of Renal and Vascular End-stage Disease (PREVEND) study.

|  | **Plasma potassium, mmol/L** | | | | |
| --- | --- | --- | --- | --- | --- |
|  | 2.3-3.4 | 3.5-3.9 | 4.0-4.4 | 4.5-4.9 | 5.0-6.3 |
| **eGFR_creatinine-cystatin C_ <60 ml/min/1.73m^2^** | |  |  |  |  |
| Person-years | 194 | 3,749 | 27,101 | 15,184 | 1,738 |
| Number of events | 5 | 21 | 152 | 90 | 10 |
| Rates* | 258 | 56 | 56 | 59 | 58 |
| Crude model | 4.81 (1.97-11.73) | 1.00 (0.63-1.58) | 1.00 (ref) | 1.06 (0.82-1.38) | 1.04 (0.55-1.98) |
| Multivariable model 1† | 2.11 (0.86-5.20) | 0.98 (0.62-1.56) | 1.00 (ref) | 0.88 (0.68-1.15) | 0.94 (0.49-1.78) |
| Multivariable model 2‡ | 2.44 (0.95-6.25) | 0.66 (0.39-1.10) | 1.00 (ref) | 0.94 (0.71-1.25) | 0.73 (0.34-1.56) |
| + Systolic blood pressure | 2.34 (0.92-5.96) | 0.65 (0.39-1.09) | 1.00 (ref) | 0.98 (0.74-1.29) | 0.80 (0.37-1.73) |
| + Aldosterone | 2.39 (0.93-6.16) | 0.69 (0.39-1.19) | 1.00 (ref) | 0.97 (0.73-1.30) | 0.80 (0.37-1.72) |
| + Plasma albumin | 2.43 (0.95-6.25) | 0.66 (0.39-1.10) | 1.00 (ref) | 0.94 (0.71-1.25) | 0.73 (0.34-1.56) |
| + Plasma magnesium | 2.10 (0.81-5.43) | 0.62 (0.36-1.05) | 1.00 (ref) | 0.94 (0.71-1.25) | 0.76 (0.35-1.62) |
| + Hs-CRP | 2.75 (1.07-7.10) | 0.70 (0.42-1.19) | 1.00 (ref) | 0.99 (0.75-1.32) | 0.72 (0.32-1.63) |
| + Smoking | 2.40 (0.94-6.16) | 0.67 (0.40-1.13) | 1.00 (ref) | 0.94 (0.71-1.24) | 0.72 (0.34-1.55) |
| + Alcohol consumption | 2.46 (0.96-6.33) | 0.66 (0.39-1.11) | 1.00 (ref) | 0.95 (0.72-1.25) | 0.73 (0.34-1.56) |
| + Education | 2.43 (0.95-6.25) | 0.66 (0.39-1.10) | 1.00 (ref) | 0.94 (0.71-1.25) | 0.73 (0.34-1.57) |
| + Race | 2.46 (0.96-6.30) | 0.66 (0.39-1.10) | 1.00 (ref) | 0.94 (0.71-1.25) | 0.73 (0.34-1.57) |
| + Type 2 diabetes | 2.42 (0.95-6.17) | 0.67 (0.40-1.12) | 1.00 (ref) | 0.91 (0.69-1.21) | 0.74 (0.35-1.59) |
| + Urinary creatinine excretion | 2.47 (0.96-6.33) | 0.66 (0.40-1.12) | 1.00 (ref) | 0.95 (0.72-1.26) | 0.73 (0.34-1.57) |
| + Plasma chloride | 2.79 (1.05-7.46) | 0.66 (0.40-1.11) | 1.00 (ref) | 0.94 (0.71-1.24) | 0.73 (0.34-1.55) |
| + BUN/creatinine ratio | 2.61 (1.02-6.67) | 0.67 (0.40-1.12) | 1.00 (ref) | 0.93 (0.70-1.23) | 0.74 (0.35-1.59) |
|  |  |  |  |  |  |
| **UAE >30 mg/24h** |  |  |  |  |  |
| Person-years | 176 | 3,800 | 27,620 | 15,629 | 1,786 |
| Number of events | 10 | 51 | 294 | 180 | 19 |
| Rates* | 568 | 134 | 106 | 115 | 106 |
| Crude model | 5.28 (2.81-9.92) | 1.26 (0.94-1.70) | 1.00 (ref) | 1.08 (0.90-1.30) | 1.00 (0.63-1.59) |
| Multivariable model 1† | 4.97 (2.64-9.37) | 1.30 (0.97-1.76) | 1.00 (ref) | 1.05 (0.87-1.26) | 0.94 (0.59-1.49) |
| Multivariable model 2‡ | 3.94 (1.97-7.85) | 0.93 (0.66-1.32) | 1.00 (ref) | 1.06 (0.87-1.29) | 1.04 (0.63-1.69) |
| + Systolic blood pressure | 3.85 (1.93-7.67) | 0.92 (0.65-1.30) | 1.00 (ref) | 1.07 (0.87-1.31) | 1.06 (0.65-1.73) |
| + Aldosterone | 3.40 (1.63-7.08) | 0.97 (0.68-1.39) | 1.00 (ref) | 1.02 (0.82-1.27) | 1.10 (0.65-1.86) |
| + Plasma albumin | 3.96 (1.98-7.90) | 0.94 (0.66-1.32) | 1.00 (ref) | 1.06 (0.87-1.30) | 1.03 (0.63-1.69) |
| + Plasma magnesium | 3.80 (1.90-7.58) | 0.94 (0.67-1.33) | 1.00 (ref) | 1.05 (0.86-1.28) | 1.04 (0.64-1.70) |
| + Hs-CRP | 4.11 (2.06-8.20) | 0.97 (0.68-1.37) | 1.00 (ref) | 1.06 (0.86-1.30) | 1.13 (0.69-1.85) |
| + Type 2 diabetes | 3.28 (1.64-6.53) | 0.92 (0.65-1.30) | 1.00 (ref) | 1.04 (0.85-1.27) | 1.05 (0.64-1.71) |
| + Smoking | 3.93 (1.97-7.85) | 0.94 (0.67-1.34) | 1.00 (ref) | 1.05 (0.86-1.28) | 1.03 (0.63-1.68) |
| + Alcohol consumption | 3.62 (1.81-7.23) | 0.93 (0.66-1.31) | 1.00 (ref) | 1.06 (0.87-1.30) | 1.05 (0.64-1.72) |
| + Education | 3.92 (1.97-7.83) | 0.93 (0.66-1.32) | 1.00 (ref) | 1.06 (0.87-1.29) | 1.04 (0.63-1.69) |
| + Race | 4.03 (2.02-8.04) | 0.94 (0.67-1.33) | 1.00 (ref) | 1.07 (0.87-1.31) | 1.05 (0.64-1.72) |
| + Urinary creatinine excretion | 3.98 (2.00-7.95) | 0.91 (0.64-1.29) | 1.00 (ref) | 1.05 (0.86-1.28) | 1.04 (0.63-1.70) |
| + Plasma chloride | 3.77 (1.85-7.68) | 0.93 (0.66-1.31) | 1.00 (ref) | 1.06 (0.87-1.30) | 1.04 (0.63-1.70) |
| + BUN/creatinine ratio | 3.97 (1.99-7.93) | 0.94 (0.66-1.32) | 1.00 (ref) | 1.06 (0.87-1.29) | 1.03 (0.63-1.69) |

Hazard ratios and 95% confidence intervals were derived from Cox proportional hazards regression models.

* Number of events divided by time at risk standardized per 10,000 person-years.

† Multivariable model 1 is adjusted for age, sex, and eGFR.

‡ Multivariable model 2 is additionally adjusted for height, weight, urinary potassium excretion, and use of diuretics.

Abbreviations: BUN, blood urea nitrogen; eGFR, estimated glomerular filtration rate; hs-CRP, high-sensitivity C-reactive protein; PREVEND, Prevention of Renal and Vascular End-Stage Disease; UAE, urinary albumin excretion.
