# Supplementary material for: Plasma potassium, diuretic use and risk of developing chronic kidney disease in a predominantly White population
Source: PLoS One. 2017 Mar 27;12(3):e0174686. doi: 10.1371/journal.pone.0174686 (PMC5367826; doi:10.1371/journal.pone.0174686)
Supplement: S3 Table — Hazard ratios and 95% confidence intervals were derived from Cox proportional hazards regression models. * Number of events divided by time at risk standardized per 10,000 person-years. † Multivariable model 1 is adjusted for age, sex, eGFR, height, weight, and urinary potassium excretion. Abbreviations: eGFR, estimated glomerular filtration rate; PREVEND, Prevention of Renal and Vascular End-Stage Disease. (DOCX) [file pone.0174686.s003.docx]

**S3 Table**. Association of plasma potassium with risk of developing chronic kidney disease stratified by use of diuretics in 5,130 participants of the Prevention of Renal and Vascular End-stage Disease (PREVEND) study.

|  | **Plasma potassium, mmol/L** | | | | |
| --- | --- | --- | --- | --- | --- |
|  | 2.3-3.4 | 3.5-3.9 | 4.0-4.4 | 4.5-4.9 | 5.0-6.3 |
| **No diuretic use** |  |  |  |  |  |
| Person-years | 80 | 2,781 | 21,469 | 12,175 | 1,291 |
| Number of events | 6 | 32 | 325 | 211 | 20 |
| Rates* | 750 | 115 | 151 | 173 | 155 |
| Multivariable model† | 7.74 (3.43-17.48) | 0.92 (0.64-1.33) | 1.00 (ref) | 1.04 (0.87-1.24) | 0.92 (0.59-1.45) |
| **Diuretic use** |  |  |  |  |  |
| Person-years | 41 | 420 | 499 | 102 | 33 |
| Number of events | 5 | 20 | 29 | 12 | 3 |
| Rates* | 1,220 | 476 | 581 | 1,177 | 909 |
| Multivariable model† | 4.32 (1.77-10.51) | 1.40 (0.88-2.22) | 1.89 (1.28-2.78) | 3.78 (2.11-6.80) | 4.79 (1.53-14.99) |

Hazard ratios and 95% confidence intervals were derived from Cox proportional hazards regression models.

* Number of events divided by time at risk standardized per 10,000 person-years.

† Multivariable model 1 is adjusted for age, sex, eGFR, height, weight, and urinary potassium excretion.

Abbreviations: eGFR, estimated glomerular filtration rate; PREVEND, Prevention of Renal and Vascular End-Stage Disease.
